# The metabogenic role of iron in chronic chagasic cardiac failure

**DOI:** 10.1590/0074-02760140461

**Published:** 2015-02

**Authors:** Carla Paixão Miranda, Fernando Antônio Botoni, Maria do Carmo Pereira Nunes, Manoel Ótavio da Costa Rocha

**Affiliations:** 1 Programa de Pós-Graduação em Infectologia e Medicina Tropical; 2 Departamento de Clínica Médica, Faculdade de Medicina, Universidade Federal de Minas Gerais, Belo Horizonte, MG, Brasil

The systematic spread of *Trypanosoma cruzi* is accompanied by an intense immune response that not only permits the control of the parasite, but also leads to a massive infiltration of mononuclear cells in the tissues affected, especially the myocardium. This leads to the local and systemic production of cytokines, chemokines and other inflammatory mediators, such as nitric oxide (Gomes et al. 2003, Miranda et al. 2014 a).

When *T. cruzi* strongly activates the cellular components within the immune system, some collateral effects could be the result (Brener 1965, Gomes et al. 2003), since the immune response seems to be greatly responsible for the tissue damage and neuronal destruction of the heart tissue observed in the acute phase of Chagas disease (Moraes-Souza 1999), contributing to one of its notable characteristics: the process of remodelling the inflamed heart characterised by a myocarditis rich in T cells and macrophages, hypertrophy and fibrosis (Bilate & Cunha-Neto 2008). During the acute phase, macrophages and dendritic cells that have made endocytosis of parasites subsequently induce a strong response of the T cells and antibodies against the antigens of *T. cruzi*. Therefore, they generate T cells that are specifically against *T. cruzi* and producers of interferon (IFN)-γ that migrate together with other leukocytes in the blood to the site of the inflammation induced by *T. cruzi* in response to the chemokines, such as CCL2, CCL3, CCL4, CCL5 and CXCL10 (Machado et al. 2010). This inflammatory response of the antibodies and T cells leads to tissue and blood parasitism control, although not completely. While in the chronic stage of the infection, probably due to the persistence of the parasite, production of the inflammatory cytokines continues because of the mononuclear cells increasing the levels of tumour necrosis factor (TNF)-α and IFN-γ in the plasma, which may be detected in infected individuals with the indeterminate forms of Chagas disease (Rocha et al. 2003).

The patients who developed chronic chagasic cardiopathy (CCC) exhibited increased levels of TNF-α and CCL2 when compared to individuals with an indeterminate form of Chagas disease (Reis et al. 1993). In addition, there was an increase in the T cells CD8^+^ and CD4^+^ in concomitance with a low production of regulatory T cells and interleukin (IL)-10 producers (Carvalho et al. 2011).

*Pathophysiological mechanisms of the alterations on the iron metabolism in heart failure (HF)* - The state of the alterations on the iron metabolism in patients with HF generally manifests itself in the chronic phase of the disease. In this context, the pathophysiologic mechanism that pervades the ischemic or dilated HF can present similarities comparable with CCC. Several factors can influence the alterations on the iron metabolism response in an individual with HF and these include, among others, genetic variations, age, gender and concomitant infections. Therefore, the alterations on the iron metabolism have obtained great prominence in recent times, due to its proven role as an indicator of hypoferremia in HF (Jankowska et al. 2010). The alterations on the iron metabolism in HF bring with it a series of cardiometabolic sequels that are worth mentioning, such as the anaemia carries a reduced oxygen supply to the tissues, generating tissue lesions, hypoxia and ischemic changes (Tang & Katz 2006). Afterwards, in response to the hypoxemia from anaemia, activation of the sympathetic nervous system begins, together with the vasoconstriction of the smaller calibre renal vessels producing renal ischaemia with the activation of a cascade of inflammatory mediators, closely related to the myocardial lesion, such as TNF-α. In this inflammatory scene, activation of the renin-angiostensin-aldosterone system (RAAS) with consequent sodium and water retention (Botoni et al. 2007). The next stage leads to the increase of cardiac remodelling. In this context, the axis that permeates through the active myocardial lesion is made up of pro-inflammatory mediators such as IL-6, IL-1 and IFN-γ which, in turn, participate in the super-expression of the peptic hepcidin hormone present in the liver with demonstrated plausible mechanisms of the metabogenic homeostasis of iron (Miranda et al. 2014b). Finally, a vicious cycle was observed that establishes between the heart and the kidney leading to the cardiorenal-anaemia syndrome.

However, studies show that IL-1, IFN-g and TNF-α act as adjuncts for the establishment of the alterations on the iron metabolism, in view that erythropoietin glycopeptide produced by the peritubular fibreblasts in the kidney's tubular cortex with a capacity to differentiate erythrocytes in mature cells and show to be diminished by the consequences of micro-lesions in the renal parenchyma, originating from the exchange between the oxidative stress-producing molecules and the pro-inflammatory cytokines that lead to cell death (Nemeth & Ganz 2009).

Alterations on the iron metabolism are common in patients with HF and are associated with the worsening of the prognostic, with a demonstrated reduction in the capacity to exercise, a poorer quality of life and increased hospitalisation. In this context, patients with CCC, since they present pathophysiologies similar to the cardiopathies, as idiopathic as ischemic, permit us to extrapolate the results of this revision of the future clinically-based investigations. Therefore, it can be concluded that, to date, no study has sought to elucidate the iron metabolism in patients with CCC and, as such, it is necessary to perform more robust clinical studies.
